# ^11^C-methionine in the diagnostics and management of glioblastoma patients with rapid early progression: nonrandomized, open label, prospective clinical trial (GlioMET)

**DOI:** 10.1186/s12885-024-12469-2

**Published:** 2024-06-15

**Authors:** Radek Lakomý, Martina Lojová, Lenka Souckova, Ludmila Hynkova, Katerina Polachova, Jiri Vasina, Regina Demlová, Alexandr Poprach, Jiri Sana, Tomas Prochazka, Martin Smrcka, Pavel Fadrus, Radim Jancalek, Iveta Selingerova, Renata Belanova, Pavel Slampa, Petr Pospisil, Tomas Kazda

**Affiliations:** 1 Department of Comprehensive Cancer Care, Masaryk Memorial Cancer Institute, 656 53 Brno, Czech Republic; 2 Department of Comprehensive Cancer Care, Faculty of Medicine, Masaryk University, 625 00 Brno, Czech Republic; 3 Department of Pharmacology, Faculty of Medicine, Masaryk University, 625 00 Brno, Czech Republic; 4 International Clinical Research Centre, St. Anne´S University Hospital Brno, 656 91 Brno, Czech Republic; 5 Department of Nuclear Medicine, Masaryk Memorial Cancer Institute, 656 53 Brno, Czech Republic; 6 Department of Neurosurgery, St. Anne’s University Hospital Brno, 656 91 Brno, Czech Republic; 7 Faculty of Medicine, Masaryk University, 625 00 Brno, Czech Republic; 8 Department of Neurosurgery, University Hospital Brno, 625 00 Brno, Czech Republic; 9 Department of Radiation Oncology, Masaryk Memorial Cancer Institute, 656 53 Brno, Czech Republic; 10 Research Centre for Applied Molecular Oncology (RECAMO), Masaryk Memorial Cancer Institute, 656 53 Brno, Czech Republic; 11 Department of Radiation Oncology, Faculty of Medicine, Masaryk University, Brno, Czech Republic; 12 Department of Medical Imaging, Masaryk Memorial Cancer Institute, 656 53 Brno, Czech Republic; 13 Present Address: Department of Clinical Trials, Masaryk Memorial Cancer Institute, Brno, Czech Republic

**Keywords:** Glioblastoma, Rapid early progression, Radiopharmaceutical, ^11^C-methionine, Clinical trial, Positron emission tomography, Radiotherapy

## Abstract

**Background:**

Glioblastoma (GBM) is the most common and aggressive primary brain cancer. The treatment of GBM consists of a combination of surgery and subsequent oncological therapy, i.e., radiotherapy, chemotherapy, or their combination. If postoperative oncological therapy involves irradiation, magnetic resonance imaging (MRI) is used for radiotherapy treatment planning. Unfortunately, in some cases, a very early worsening (progression) or return (recurrence) of the disease is observed several weeks after the surgery and is called rapid early progression (REP). Radiotherapy planning is currently based on MRI for target volumes definitions in many radiotherapy facilities. However, patients with REP may benefit from targeting radiotherapy with other imaging modalities. The purpose of the presented clinical trial is to evaluate the utility of ^11^C-methionine in optimizing radiotherapy for glioblastoma patients with REP.

**Methods:**

This study is a nonrandomized, open-label, parallel-setting, prospective, monocentric clinical trial. The main aim of this study was to refine the diagnosis in patients with GBM with REP and to optimize subsequent radiotherapy planning.

Glioblastoma patients who develop REP within approximately 6 weeks after surgery will undergo ^11^C-methionine positron emission tomography (PET/CT) examinations. Target volumes for radiotherapy are defined using both standard planning T1-weighted contrast-enhanced MRI and PET/CT. The primary outcome is progression-free survival defined using RANO criteria and compared to a historical cohort with REP treated without PET/CT optimization of radiotherapy.

**Discussion:**

PET is one of the most modern methods of molecular imaging. ^11^C-Methionine is an example of a radiolabelled (carbon 11) amino acid commonly used in the diagnosis of brain tumors and in the evaluation of response to treatment. Optimized radiotherapy may also have the potential to cover those regions with a high risk of subsequent progression, which would not be identified using standard-of-care MRI for radiotherapy planning. This is one of the first study focused on radiotherapy optimization for subgroup of patinets with REP.

**Trial Registration:**

NCT05608395, registered on 8.11.2022 in clinicaltrials.gov; EudraCT Number: 2020–000640-64, registered on 26.5.2020 in clinicaltrialsregister.eu. Protocol ID: MOU-2020–01, version 3.2, date 18.09.2020.

## Introduction

### Background and rationale {6a}

Compared to other oncological diagnoses, primary brain tumours are less common, with an incidence of 8/100,000 population. Unfortunately, the most aggressive tumors, glioblastomas (GBM), account for approximately half of all primary malignant brain tumors in adults. Due to their biological behaviour, these tumours are ranked among the most difficult-to-treat diseases and therefore represent a serious health problem despite the relatively low incidence. Despite advances in the complex oncological treatment of gliomas, treatment results remain unsatisfactory [1, 2].

The reported median survival of 14–17 months with a five-year survival of 10% is observed mainly in patients with favourable prognostic factors who undergo complete adjuvant oncological therapy [3, 4]. Despite all efforts, the median overall survival has only increased by a few months over the past thirty years. For this reason, further research and development of new therapeutic procedures are necessary with the aim of ensuring better disease control and prolonging overall survival [5].

Clinical studies evaluating the role of modern targeted therapy and immunotherapy have not yet demonstrated higher effectiveness of this treatment strategy, and the results of GBM treatment have thus not fundamentally changed. The only small advance in the last 15 years has been treatment using alternating electric fields emitted from electrodes taped to the scalp (Optune). However, the broader availability of this method is limited by the enormously high cost, patient motivation, and some debatable issues in the clinical trials performed [6, 7].

The current standard of care for GBM is based on a multimodal approach combining the maximum possible and safe surgery, postoperative radiotherapy (RT), and chemotherapy (CHT) with alkylating cytostatic temozolomide (TMZ). Adjuvant therapy is usually started within 4 to 6 weeks after surgery, during which the patient recovers from surgery, and all necessary procedures (multidisciplinary indication committee, preparation of radiation plan, etc.) take place before further oncological treatment. The standard postoperative management of newly diagnosed patients with GBM has remained unchanged since the published results of the EORTC 26981–22981/NCIC CE3 trial (Stupp regimen), which completed patient recruitment as early as 2002 and published the findings in 2005. In this protocol, TMZ (75 mg/m^2^) is administered on days 1 to 42 concurrently with RT (60 Gy), followed by TMZ alone (150 to 200 mg/m^2^) on days 1–5 in six consecutive 4-week cycles. Coadministration of RT and TMZ improved survival from 12.1 months (with RT alone) to 14.6 months (with the addition of TMZ) (3). Due to a certain stagnation in the treatment of GBM in recent years, intensive research at all levels is necessary to improve patients' perspectives on this unfavourable diagnosis.

The Perry accelerated regimen [8] is a treatment option for patients aged ≥ 65 years and those for whom long conventional RT (60 Gy in six weeks) combined with chemotherapy is considered unsuitable by their treating physicians. The total RT dose for this accelerated regimen is 40.05 Gy, administered in 15-day fractions over three weeks.

Concomitant TMZ is administered here at a dose of 75 mg/m^2^ per day for 21 consecutive days from the start of RT until its termination. As with Stupp's regimen, adjuvant TMZ alone follows at 150 to 200 mg/m^2^ daily for five consecutive days of a 28-day cycle, up to 12 cycles or until disease progression. For patients who are not candidates for concomitant chemoradiotherapy (regardless of age), individual adjuvant treatment is indicated as standard, usually accelerated RT alone (total dose 30–50 Gy in a 10–20-day dose, for example, fractionation regimens of 10 × 3.0 Gy, 10 × 3.4 Gy, 15 × 2.67 Gy, 20 × 2.5 Gy and others), followed in some patients by TMZ palliative chemotherapy based on the actual overall condition of the patient.

#### Glioblastoma as a progressive disease

In connection with the greater availability of magnetic resonance (MR), it is possible to optimize the planning of radiotherapy according to the findings of the so-called planning MR for an increasing number of patients. It is an MR study performed immediately (approximately a few days) before the start of radiotherapy. Following the performance of this MR study (typically approx. 4–5 weeks after surgery), a new phenomenon called very rapid progression (REP, rapid early progression) is described. The REP diagnosis is based on comparing early postoperative MR findings (usually within 48–72 h after surgery) and planning MR before radiotherapy (preRT).

Retrospectively comparing early postoperative MR with preRT MR performed approximately 30 days later, Farace et al. described 30% of preRT MR with signs strongly suggestive of tumour progression during this period [9]. An even more significant proportion of patients with documented REP was described by Palmer et al., where up to 52% progressed before starting adjuvant therapy without any effect of waiting time to start RT (median time from resection to RT was 32.5 vs. 33 days in patients with REP vs. without REP (*p* = 0.337) [10]. These results are consistent with our retrospective analysis of 90 patients with GBM treated between 2014 and 2017, where 51% of patients had suspected REP at planning preRT MR [11]. These patients represent a subset with particularly aggressive GBM requiring further intensive research and possibly treatment modification.

According to these and several other retrospective analyses, it was confirmed that the presence of early progression on planning MR was associated with a more aggressive form of GBM and worse overall survival [12–14]. A higher risk can, of course, be expected in patients after nonradical resections [14]. It is still unclear what further influences the prognosis of patients with early progression. Palmer et al. even described significantly worse survival in patients with unmethylated MGMT (O6-methylguanine-DNA methyl-transferase), the methylation of MGMT is considered a marker of higher chemosensitivity [10]. Analysis of other potential biomarkers still needs to be improved. Due to a large number of patients with REP and the retrospective nature of the studies carried out thus far, it is essential to analyse this more aggressive subgroup of tumors prospectively and try to influence the negative course of the disease more clinically.

The optimal treatment approach for patients with REP needs to be determined. It is unclear whether it is better to indicate repeat surgery for recurrence, choose accelerated RT regimens with or without concurrent chemotherapy, or directly administer more aggressive and intensive chemotherapy with a combination of alkylating cytostatics if MGMT methylation is present [15]. Treatment of these patients with REP currently does not differ from that of patients without REP; if so, it is a purely individual approach. These patients distorted the results of previous clinical trials where routine MR examination was not performed before RT. Currently, these patients are usually excluded from clinical trials.

Moreover, current clinical trials often randomize patients until after concomitant chemoradiotherapy if there is no progression on follow-up MR after chemoradiotherapy. REP on planning MR is a significant negative prognostic factor that should be a stratification factor in future clinical trials. In general, the aggressive character of GBM is manifested by its microenvironment, the molecular background of glioma cells, or the miRNA profile, which may be essential in GBM oncogenic signalling and has the potential to serve as a biomarker of this disease and a new therapeutic target in oncology [16–22].

#### Radiotherapy in the treatment of glioblastoma

Due to the almost zero risk of developing distant metastases, and on the contrary, due to the nearly 100% risk of developing local recurrence in the brain, in the case of GBM, great emphasis is placed on local treatment methods, i.e., surgery and radiation. Radiotherapy has experienced a stormy development in the last decade due to improved computer technology, greater availability of imaging methods, and more advanced radiotherapy systems [7, 23, 24]. A crucial part of radiotherapy treatment is correctly determining the target area for the prescribed radiation dose. In the case of brain tumours, the gold standard for defining target volumes is MR imaging. Recently, the importance of additional imaging methods, especially PET imaging, for the closer characterization of gliomas has been emphasized [24, 25].

#### ^11^C-Methionine PET in the imaging of glioblastoma

Positron emission tomography is currently the most dynamically developing area for functional brain imaging. It is used both pretreatment and as part of the follow-up of GBM patients after treatment. The development of PET diagnostics is aimed not only at constructing more sensitive and robust PET scanners but also at introducing new radiopharmaceuticals, especially in centres with their own cyclotron.

Fluorodeoxyglucose (2-deoxy-2-[18F]fluoro-D-glucose, FDG) is the most commonly used radiopharmaceutical in PET/CT diagnostics in general. However, it may not provide optimal results for brain examination mainly due to the high metabolic activity in the physiological brain tissue, the image background. That is why other radiopharmaceuticals are used in the targeted diagnosis of brain tumors, most often different amino acid derivatives. Of these, [18F]-O-(2-fluoroethyl)-L-tyrosine (fluoroethyltyrosine, FET) and L-(S-methyl-[^11^C])-methionine (methionine, MET) play the most important roles. Carbon ^11^-labelled methionine is historically the most frequently used drug for glioma imaging.

Amino acid (AA) tracers, including FET and MET, naturally cross the blood–brain barrier through neutral amino acid transport mechanisms. Unlike naturally occurring methionine, the artificially prepared amino acid FET is not subsequently incorporated into protein fractions [26, 27]. Their transport and incorporation into proteins are increased in tumour tissue. However, it is still being determined which of these mechanisms is more critical for PET imaging. Methionine is also incorporated into fats and nucleic acids; this incorporation is less significant for imaging brain tumours. Studies have confirmed the correlation between the intensity of MET accumulation, tumour grading according to WHO and proliferative activity assessed by the Ki-67 index [28] and the density of microvascularization [29]. Methionine is incorporated to an increased extent even in low-grade gliomas [30], while its accumulation is low in the background of normal brain tissue. Since the transport of AA tracers does not depend on the blood‒brain barrier violation, the accumulation of AA tracers can be detected even in tumours that do not show contrast saturation and enhancement during MR examination.

MET PET is more suited than MR to accurately delineate the target volume for radiotherapy and determine the extent of viable tumours. Conversely, MR shows the overall extent, including pathological changes associated with the tumour, such as oedema in the immediate vicinity. The widespread use of MET PET is most hindered by its short half-life, so the use of MET is limited only to centres with their cyclotron and the possibility of its production. In patients with REP glioblastoma, it is necessary to start oncological treatment as quickly as possible. It is, therefore, unethical to wait for the delivery of other commercially available radiopharmaceuticals labelled with fluorine 18 (18F-FET, 18F-FLT, 18F-DOPA, etc.), which have a longer half-life. They are also available in PET centres without a cyclotron, but only on predetermined and planned production days. In complex neuro-oncology centres, the possibility of individual preparation of MET is thus an opportunity to benefit patients with REP, the extraordinarily aggressive glioblastomas.

In addition to the subjective assessment, the evaluation of the PET image is refined by quantitative measurement. For this quantitative measurement in clinical practice, drawing a suitable area of interest and software evaluation of the SUVmax parameter ("maximum standardized intensity of accumulation") are usually used. The assessment of the SUVavg ("average value of the standardized intensity of accumulation") is chosen less often. Nevertheless, it is influenced by the specific location of the area of interest, with more significant variability between evaluators. Therefore, SUVavg is less suitable for standardized assessment in studies.

The area of interest can be either circular in the plane for one selected slice, 2D-ROI, or spherical in space, 3D—VOI. The two-dimensional region of interest is also very strongly influenced by the choice of a particular slice for assessment and is also burdened by low reproducibility and high interrater variability. For our evaluation, the measurement of SUVmax in a spatial 3D-VOI defined with a size of 1.5 cm was chosen to minimize variability.

The ratio of the radiopharmaceutical accumulation in the tumour relative to the background ("tumour-to-background" ratio, T/B) is used to differentiate the areas of the physiological and pathological intensity of accumulation. There is no definite recommendation on how to choose the background, and even any recommendations may be influenced by the possibilities of a specific patient. There are also no exact values of the T/B ratio defined as a threshold for distinguishing tumour tissue from physiological tissue. According to available literature data, values between 1.3 and 1.7 are usually chosen.

### Explanation for choice of comparator {6b}

This study is an open-label prospective clinical trial. The experimental drug ^11^C-methionine PET/CT will be administered to all patients recruited to this study and compared to the cohort consisting of a historical group of GBM patients with REP collected in the period 2014–2018.

### Objectives {7}

Patients diagnosed with GBM who develop REP before starting adjuvant radiotherapy have an abysmal prognosis. The optimal treatment for these patients has yet to be discovered, and no prospective clinical evaluation has been performed. We assume that the lesion detected on MR is not the only area of REP development. It may be MET PET that can reveal other areas of the aggressive part of the tumour, and the subsequent RT can then be better planned to increase the time to progression in such a cohort of patients. Therefore, the purpose of this study was to refine the diagnosis in patients with GBM with proven REP and subsequent optimized planning of the following treatment procedure. Chemotherapy will be administered as standard treatment to all patients.

### Trial design {8}

The GlioMET study is an investigator-initiated study. This clinical trial is a prospective, monocentric, open-label, nonrandomized clinical study with a parallel assignment initiated in June 2020.

**Fig. 1:**
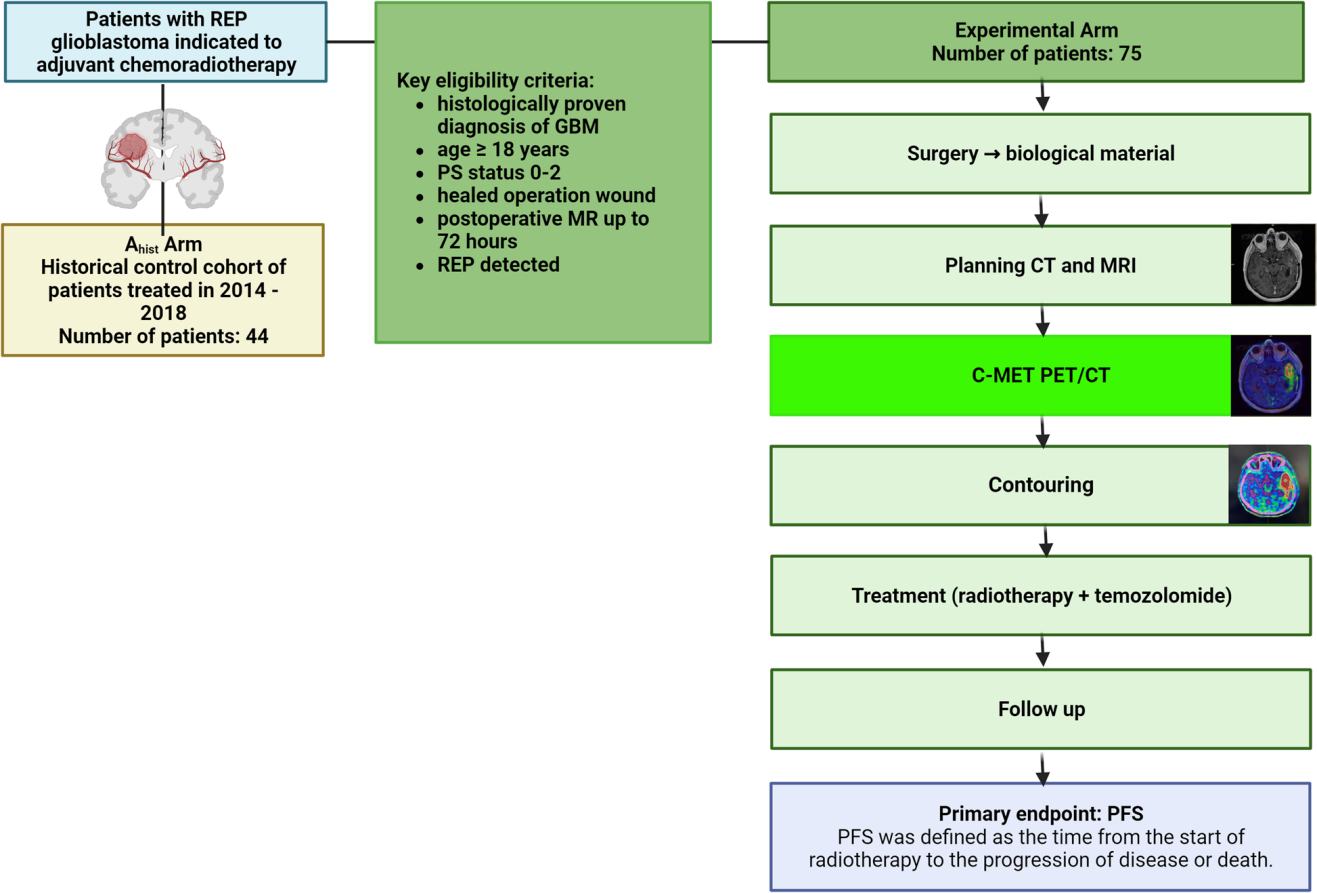
The Schematic overview of the GlioMET study design. A_hist_ Arm – a historical arm. C-MET—carbon radiolabelled methionine. CT – computer tomography. GBM—glioblastoma multiforme. MRI – magnetic resonance imaging. PET/CT—positron emission tomography/computer tomography. PFS – progression-free survival. PS – performance status. REP—rapid early progression

The main aim of the clinical trial is to improve the progression-free survival of patients with GBM and REP via optimizing of diagnostic and treatment procedures. The experimental drug ^11^C-methionine PET/CT will be administered to all patients recruited to this study and compared to the cohort consisting of a historical group of patients collected in the period 2014–2018. The setting is a confirmatory framework against the historical cohort. A schematic overview of the design of the study is presented in Fig. 1.

## Methods: participants, interventions, and outcomes

### Study setting {9}

The GlioMET study is a monocentric study. The recruitment period began in October 2020. Patient recruitment in the Czech Republic is planned to be completed in February 2024.

Masaryk Memorial Cancer Institute in the Czech Republic, the coordinating centre for the study, is responsible for educating and training research staff, tracking participants’ enrolment, mathematical analysis, monitoring, pharmacovigilance, data management, and reporting of the study. The control cohort consisted of a historical group of patients collected in the period 2014–2018. Data are collected only from the Czech Republic.

### Eligibility criteria {10}

A patient may be included in the clinical trial if they meet all of the following criteria:

1. The participant is a person with a histologically proven diagnosis of glioblastoma (GBM) according to WHO 2016.
2. The participants are male or female and aged 18 years or older.
3. Performance status (PS) according to ECOG (Eastern Cooperative Oncology Group) 0–2.
4. Healed operation wound.
5. Postoperative MR up to 72 h.
6. Indication for adjuvant chemoradiotherapy.
7. The participants must express his/her informed consent and sign the form before the screening period.
8. Rapid early progression was detected.
9. The participants must achieve the following values of laboratory parameters in the peripheral blood during the screening period:
  neutrophils (total count) ≥ 1500/mm.3
  platelets (total count) ≥ 100,000/mm.3
  hemoglobin ≥ 9.0 g/dL
  serum creatinine ≤ 1.5 × of upper limit of normal (ULN)
  total bilirubin ≤ 1.5 × ULN, unless documented Gilbert's syndrome, for which bilirubin ≤ 3 × ULN is permitted
  AST/ALT ≤ 3 × ULN

The exclusion criteria were as follows:

1. Prior brain surgery.
2. Prior brain radiotherapy.
3. The history of active/currently treated cancer (solid tumor) was as follows: nonmelanoma skin cancer, in situ bladder carcinoma, in situ gastric cancer, in situ colorectal carcinoma, in situ cervical carcinoma, and in situ breast cancer.
4. Any systemic disease or health condition that might pose a risk for anticancer therapy and imaging techniques (MRI, MET PET).
5. Patients must not have substance abuse disorders that would interfere with cooperation with the requirements of the trial.
6. Patients must not have any evidence of ongoing (active) infection (HIV, hepatitis A, B, C).
7. Pregnant and/or breastfeeding women.
8. Patients who disagreed and refused to sign an informed consent form.

### Intervention description {11a}

The intervention is the radiopharmaceutical application performing ^11^C-methionine PET examination. The activity of the ^11^C-methionine applied for each patient will be 300 MBq (± 50 MBq) of ^11^C-methionine. The preparation of ^11^C-methionine for a specific patient is provided by a lab worker only be performed with written consent. ^11^C-MET will be sufficiently hydrated, meaning that they have not consumed any food for at least 4 h prior to application. To obtain the best quality images and to reduce radiation exposure to the bladder, the patient will be asked to drink plenty of fluids and void before the PET scan. After a PET scan, the bladder should be emptied as often as possible. If patient sedation is needed, it will be started approximately 20–60 min before the start of the examination. The application of the radiopharmaceutical will be carried out after ensuring venous access with an intravenous cannula. Finish the application by rinsing with 20 ml of physiological solution. The patient will be monitored for 2 h after the application of the radiopharmaceutical due to the possibility of a hypersensitivity reaction.

### Criteria for discontinuing or modifying allocated interventions {11b}

The clinical trial will be interrupted or prematurely terminated for the following reasons:

1. In the case of serious adverse events, the resulting benefit-risk ratio is unacceptable.
2. In the event that other comparable clinical evaluations reveal an unacceptable level of risk for the evaluation subjects. The sponsor reserves the right to interrupt or prematurely end the clinical trial at the centre in the event that:
  (i) the safety of the subjects would be significantly compromised,
  (ii) sufficient recruitment would not be achieved,
  (iii) there would be repeated deviations from the protocol on the part of the investigating centre.

### Strategies to improve adherence to interventions {11c}

Training before initialization ensures compliance during the whole study. If there are doubts, a chief investigator is designated to address them.

### Relevant concomitant care permitted or prohibited during the trial {11d}

The modification consists of changing the irradiation plan. Two chemoradiotherapy regimens of concomitant chemoradiotherapy with temozolomide are permissible at the discretion of the attending physician [31–33]:

#### Regime according to Stupp:

RT 60 Gy/30 fr. each 2.0 Gy (6 weeks, Mon-Fri) + temozolomide 75 mg/m^2^ D1-42 including weekends, then adjuvant temozolomide 150–200 mg/m^2^ D1-5, 28-day interval, six cycles with the possibility of extension to a total of 12 cycles (if no more than low-grade adverse effects occur and if a benefit is demonstrated) or until progression or toxicity.

#### Perry's regimen

RT 40.05 Gy/15 fr. each 2.67 Gy (3 weeks, Mon-Fri) + temozolomide 75 mg/m^2^ D1-21 including weekends then adjuvant temozolomide 150–200 mg/m^2^ D1-5, interval 28 days, max. 12 cycles or until progression or toxicity.

The choice of regimen is at the discretion of the attending physician. In general, the Perry regimen is used for frail and elderly patients, and these patients would probably not be able to handle the classic long Stupp regimen. It is a modified Stupp regimen, where there is a shorter (3-week) concomitant CHT/RT with 40.05 Gy instead of 6 weeks with 60 Gy, followed by classical adjuvant 150–200 mg/m^2^, but up to 12 cycles of temozolomide.

### Outcomes {12}

Primary Outcome Measures of the clinical trial are to demonstrate the progression-free survival (PFS) in the 11C-methionine PET/CT arm as Experimental arm vs. PFS in the historical patient cohort as an arm Ahist. PFS was defined as the time from the start of radiotherapy to the progression of disease or death [Time Frame: 44 months].

The secondary outcomes are the following:

1. Rapid-Early-Progression incidence [Time Frame: 38 months]
  Prospective evaluation of the incidence of Rapid-Early-Progression (REP) on planning MR in patients with GBM indicated for adjuvant chemoradiotherapy.
2. Overall survival (OS) in the Experimental Arm vs. Arm Ahist [Time Frame: 44 months]
  OS in the Experimental Arm compared to the historical retrospective group (arm Ahist). OS = time from surgery to death related to glioblastoma multiforme.
3. Biomarkers I [Time Frame: 38 months].
  Immunohistochemical analysis of GFAP, Ki-67, IDH1, ATRX, and PDL1. GFAP: positive/negative/focal positive Ki-67, IDH1: positive/negative, ATRX loss of expression: positive/negative, PDL1 protein expression: positive/negative.
4. Biomarker II [Time Frame: 38 months]
  Mutational status of TERT and IDH2: mutated/wild-type.
5. Biomarkers III [Time Frame: 38 months]
  Sanger sequencing of IDH1 in IDH1 IHC-positive patients: mutated/wild-type.
6. Biomarkers IV [Time Frame: 38 months]
  MGMT promoter methylation status: ≥ 25% = methylated/ < 25% = unmethylated.
7. Biomarkers V [Time Frame: 38 months]
  1 p/19 q codeletion: positive/negative
8. Pattern-of-failure analysis [Time Frame: 44 months]
  Evaluation of spatial patterns of failure (PoF; central (V95% ≥ 95%)/in-field (80% ≤ V95%)/marginal (20% ≤ V95% < 80%)/distant (V95% < 20%)) in patients with REP (the Experimental Arm) compared to the historical retrospective group (Arm Ahist). In the case of multifocal progression, each PD is evaluated independently, i.e., the patient could have one central and one distal PD. In subsequent statistical analysis, each PD was evaluated independently. PoF could be performed even in deceased subjects if MR describing the progression is available.
9. Quality of Life assessment using the standardized European Organization for Research and Treatment of Cancer (EORTC) questionnaire QLQ-C30 [Time Frame: 44 months]
  Quality of life will be assessed using a standardized questionnaire: the European Organization for Research and Treatment of Cancer Quality of Life Questionnaire C30 v.3.0 during the screening period, visits 2, 4, 6, 8, 10, and 12 (End-Of-Treatment) and during the follow-up period every 3 months (± 1 week) up to progression of the disease.
10. Quality of Life assessment using the standardized European Organization for Research and Treatment of Cancer (EORTC) Quality of Life questionnaire-BN20 [Time Frame: 44 months]
  Quality of life will be assessed using a standardized questionnaire: additional module Brain Cancer European Organization for Research and Treatment of Cancer Quality of Life questionnaire BN20 during the screening period, visits 2, 4, 6, 8, 10, and 12 (End-Of-Treatment) and during the follow-up period every 3 months (+/- 1 week) up to the disease.

### Participant timeline {13}

The schedule of enrolment, intervention and visits is summarized in Tables 1 and 2. The duration of the study participation is 44 months (recruitment 38 months + 6 months follow-up).

**Table 1:** Participant timeline for the regimen according to Stupp

4.3.1.Stupp regimen	Screening	CHEMO RADIO THERAPY	Visit 1	Visit 2	Visit 3	Visit 4	Visit 5	Visit 6	Visit 7	Visit 8	Visit 9	Visit 10	Visit 11	Visit 12 (EoT)	Follow-up until PD	EoS Visit
Days / Months	**max. 14 D**	**D1-D42** **(M 1–2)**	**D42 + 1 M** (± 1 tweek)	** + 1 M**	** + 1 M**	** + 1 M**	** + 1 M**	** + 1 M**	** + 1 M**	** + 1 M**	** + 1 M**	** + 1 M**	** + 1 M**	** + 1 M**	**Every 3 M** (± 1 tweek)	**PD**
Inclusion/exclusion criteria	X															
Informed consent	X															
Anamnesis^a^	X															
Pregnancy test (serum/urine)	x*	xx*	x	x	x	x	X	x	x	x	x	x	x			
HIV, HBV, HCV test	X**															
Physical and laboratory examination^b^	X**	x***	x	x	x	x	x	x	x	x	x	x	x	x		
ECOG Performance status	X	x	x	x	x	x	x	x	x	x	x	x	x	x		
Extent of surgery^c^	x															
^11^C-MET PET/CT^d^	x															
Quality of life questionnaires^e^	x			x		x		x		x		x		x	x	
MR and RANO evaluation^f^				x (á 3 months)												
Molecular markers^g^	*To be performed during the patient's participation in the study*															
Radiotherapy **(start max. 8 weeks since surgery and max. 2 weeks after planning MR)**		x														
Chemotherapy **(start max. 8 weeks since surgery and max. 2 weeks after planning MR)**		x	x	x	x	x	x	X	x	x	x	x	x	x		
Adverse events		x	x	x	x	x	x	X	x	x	x	x	x	x	x	x
PoF analysis^h^																x

The description of performed examinations:

^*^The pregnancy test will be performed at the center (1. As part of the screening phase before 11C-methionine applications and 2. Before the 1st application of concomitant chemoradiotherapy)

^**^Laboratory analysis incl. tests for HIV, HBV and HCV will be performed in the screening phase if they have not been performed in the previous 5 days

^***^Laboratory analysis (blood count + differential + biochemistry) will be performed every week as part of CHRT. Physical examination will be performed on D1 and D21 (if not performed in the previous 5 days)

^a^Anamnesis

Collection of anamnestic data will take place following standard clinical practice. Taking a family history will focus on the familial occurrence of malignancies and genetic syndromes. As part of the personal anamnesis, pre-existing severe diseases will be recorded even at the time of diagnosis; a pharmacological anamnesis and information on allergies will automatically be included. Data will also be collected regarding substance abuse and social and work anamnesis

^b^Physical and laboratory examination

Physical examination + neurological examination + height/weight/vital functions: BSA calculation, blood pressure, pulse, respiratory rate, body temperature;

Performance status: according to ECOG 0–2; Laboratory examination: blood count including differential count, Na, K, Cl, urea, creatinine, glucose, total bilirubin, ALT, AST, ALP, amylase, LD, albumin, total protein, CRP

^c^Scope of operation

Evaluation according to postoperative MR (performed within 72 h after surgery):

Gross Total Resection: complete macroscopic resection

Subtotal Resection: resection of ≥ 90% of the tumor

Partial Resection: resection of < 90% of tumor (biopsy only = exclusion criterion)

^d11^C-MET PET/CT

^11^carbon radiolabelled methionine positron emission tomography/computer tomography

^e^Quality of life questionnaire

These are standardized questionnaires assessing the quality of life of oncology patients, according to the EORTC. Official versions of the questionnaires in Czech are available. These questionnaires can be completed at home. The basic questionnaire (EORTC QLQ-C30 Czech) contains 30 questions with the possibility of answering a degree of four points or a seven-point Likert scale. Part of the quality of life questionnaires is also a module of an additional 20 questions optimized to assess the quality of life of neuro-oncology patients

^f^Magnetic Resonance Imaging and assessment of RANO criteria

^g^Molecular markers

Immunohistochemical labeling of GFAP, Ki-67, IDH1 and ATRX, PDL1 protein expression by immunohistochemistry, mutational status of TERT and IDH2 genes and then IDH1 in immunohistochemically positive patients by Sanger sequencing, methylation status of the MGMT promoter and 1p/19q codeletion

^h^PoF analysis

Spatial evaluation of recurrence in relation to the area irradiated with a high dose of radiation (patterns of failure, PoF) is performed by a radiotherapist. MR defining progression according to RANO (MRRANO) criteria is used to analyze PoF, specifically T1-weighted sequence with contrast medium administration

Standard image fusion is performed in the radiotherapy planning system: MRRANO is registered to the original planning CT. The fusion is focused on the calf area (preferential choice of Hounsfield HU units in the bone band)

**Table 2:** Participant timeline for the regimen according to Perry

4.3.2. Perry regimen	Screening	CHEMO RADIO THERAPY	Visit 1	Visit 2	Visit 3	Visit 4	Visit 5	Visit 6	Visit 7	Visit 8	Visit 9	Visit 10	Visit 11	Visit 12 (EoT)	Follow-up until PD	EoS Visit
Days / Months	**max. 14 D**	**D1-D21** **(M 1–2)**	**D21 + 1 M** (± 1 week)	** + 1 M**	** + 1 M**	** + 1 M**	** + 1 M**	** + 1 M**	** + 1 M**	** + 1 M**	** + 1 M**	** + 1 M**	** + 1 M**	** + 1 M**	**Every 3 M** (± 1 week)	**PD**
Inclusion/exclusion criteria	x															
Informed consent	x															
Anamnesis^a^	x															
Pregnancy test (serum/urine)	x*	x*	x	x	x	x	x	x	x	x	x	x	x			
HIV, HBV, HCV test	X**															
Physical and laboratory examination^b^	X**	x***	x	x	x	x	x	x	x	x	x	x	x	x		
ECOG Performance status	x	x	x	x	x	x	x	x	x	x	x	x	x	x		
Extent of surgery^c^	x															
^11^C-MET PET/CT^d^	x															
Quality of life questionnaires^e^	x			x		x		x		x		x		x	x	
MR and RANO evaluation^f^				x (á 3 months)												
Molecular markers^g^	*To be performed during the patient's participation in the study*															
Radiotherapy **(start max. 8 weeks since surgery and max. 2 weeks after planning MR)**		x														
Chemotherapy **(start max. 8 weeks since surgery and max. 2 weeks after planning MR)**		x	x	x	x	x	x	x	x	x	x	x	x	x		
Adverse events		x	x	x	x	x	x	x	x	x	x	x	x	x	x	x
PoF analysis^h^																x

The description of performed examinations:

^*^The pregnancy test will be performed at the center (1. As part of the screening phase before 11C-methionine applications and 2. Before the 1st application of concomitant chemoradiotherapy)

^**^Laboratory analysis incl. tests for HIV, HBV and HCV will be performed in the screening phase if they have not been performed in the previous 5 days

^***^Laboratory analysis (blood count + differential + biochemistry) will be performed every week as part of CHRT. Physical examination will be performed on D1 and D21 (if not performed in the previous 5 days)

^a^Anamnesis

Collection of anamnestic data will take place following standard clinical practice. Taking a family history will focus on the familial occurrence of malignancies and genetic syndromes. As part of the personal anamnesis, pre-existing severe diseases will be recorded even at the time of diagnosis; a pharmacological anamnesis and information on allergies will automatically be included. Data will also be collected regarding substance abuse and social and work anamnesis

^b^Physical and laboratory examination

Physical examination + neurological examination + height/weight/vital functions: BSA calculation, blood pressure, pulse, respiratory rate, body temperature;

Performance status: according to ECOG 0–2; Laboratory examination: blood count including differential count, Na, K, Cl, urea, creatinine, glucose, total bilirubin, ALT, AST, ALP, amylase, LD, albumin, total protein, CRP

^c^Scope of operation

Evaluation according to postoperative MR (performed within 72 h after surgery):

Gross Total Resection: complete macroscopic resection

Subtotal Resection: resection of ≥ 90% of the tumor

Partial Resection: resection of < 90% of tumor (biopsy only = exclusion criterion)

^d11^C-MET PET/CT

^11^carbon radiolabelled methionine positron emission tomography/computer tomography

^e^Quality of life questionnaire

These are standardized questionnaires assessing the quality of life of oncology patients, according to the EORTC. Official versions of the questionnaires in Czech are available. These questionnaires can be completed at home. The basic questionnaire (EORTC QLQ-C30 Czech) contains 30 questions with the possibility of answering a degree of four points or a seven-point Likert scale. Part of the quality of life questionnaires is also a module of an additional 20 questions optimized to assess the quality of life of neuro-oncology patients

^f^Magnetic Resonance Imaging and assessment of RANO criteria

^g^Molecular markers

Immunohistochemical labeling of GFAP, Ki-67, IDH1 and ATRX, PDL1 protein expression by immunohistochemistry, mutational status of TERT and IDH2 genes and then IDH1 in immunohistochemically positive patients by Sanger sequencing, methylation status of the MGMT promoter and 1p/19q codeletion

^h^PoF analysis

Spatial evaluation of recurrence in relation to the area irradiated with a high dose of radiation (patterns of failure, PoF) is performed by a radiotherapist. MR defining progression according to RANO (MRRANO) criteria is used to analyze PoF, specifically T1-weighted sequence with contrast medium administration

Standard image fusion is performed in the radiotherapy planning system: MRRANO is registered to the original planning CT. The fusion is focused on the calf area (preferential choice of Hounsfield HU units in the bone band)

The modification consists of changing the irradiation plan.

Two chemoradiotherapy regimens of concomitant chemoradiotherapy with temozolomide are permissible at the discretion of the attending physician.

Stupp's regimen can be extended to 12 cycles of adjuvant temozolomide due to the unification of the possible length of administration of adjuvant temozolomide itself and thus minimizing the occurrence of bias in the length of adjuvant treatment between younger and older/frail groups in the clinical study.

Since 2005, when temozolomide was registered based on the study with 6 cycles of adjuvant temozolomide, a number of phase 3 studies were conducted, where it was possible to extend this adjuvant period up to 12 cycles. On the other hand, in patients without REP, extension of adjuvant chemotherapy up to 12 cycles is currently not recommended [2].

### Sample size {14}

A historical retrospective cohort included 44 patients with REP who were indicated for adjuvant chemoradiotherapy (Arm Ahist). The median PFS in this cohort was 4.9 months. Assuming that the time distribution in the Experimental Arm follows a proportional hazard model, a significance level of 5%, and a power of 80%, 65 events are needed to detect a hazard ratio of 1.65 (i.e., median PFS in the Experimental Arm of 8 months). With an assumed recruitment of 38 months and a follow-up duration of six months, 67 patients are needed based on the historical cohort. With the expected screening failure rate of 5% and a dropout rate of 5%, approximately 75 patients with confirmed REP should be enrolled.

### Recruitment {15}

Recruitment of the patients has been carried out since October 2020. During the first four years, all planned patients will be recruited. The patients were recruited only at Masaryk Memorial Cancer Institute in Brno, Czech Republic.

The patient will be informed about the study during the visit, during which initial eligibility for the study will be determined, and the research staff will explain the study protocol and design.

## Methods: assignment of interventions: allocation

### Sequence generation {16a}, Concealment mechanism {16b}

The GlioMET study is nonrandomized, and the assignment of interventions is not applicable.

### Implementation {16c}

The study investigator will enroll the participants. All study visits will be scheduled to coincide with the regimen according to Stupp or Perry.

### Blinding (masking) {17a}

Not applicable. This study is not blinded; it is open-label.

## Methods: data collection, management and analysis

### Data collection methods {18a}

The study collected demographic and baseline characteristics from medical records and electronic medical records, including age, sex, type of admission, and baseline characteristics such as body weight, body height, patient history, and pharmacological history. The results of PET/CT scans and physical examinations are documented in the electronic medical records and entered by the trial investigator in the electronic Case Report Form (eCRF).

### Plans to promote participant retention and complete follow-up {18b}

The study site will make every reasonable effort to follow the participant for the entire study period. Study site staff members will be responsible for developing and implementing local standard operating procedures to achieve maximal follow-up.

### Data management {19}

The study data are entered online in an internet-based database (RedCap®) and collected from medical records. The study staff members had access to the medical records of the patients. The investigators will be responsible for screening patients, obtaining informed consent, collecting study data, and entering it into the eCRF. The statistician will analyse the study data in cooperation with the principal investigator. The data will be stored for 15 years after completion of the study and then destroyed. To promote data quality, the eCRFs of each participant will be reviewed by another member of the study team as a monitor.

## Statistical methods

### Statistical methods for primary and secondary outcomes {20a}

All data will be described for each arm separately and for the entire group of patients. The appropriate descriptive statistics will be used, i.e., counts and percentages for categorical characteristics and median and interquartile range (IQR) or mean and standard deviation (SD) for continuous characteristics. Depending on the data type, common statistical tests such as Fisher's exact test or chi-square test for comparing categorical variables and nonparametric Mann‒Whitney test or Kruskal‒Wallis test for comparing continuous variables or their parametric alternatives if the assumption of data normality is met will be used for comparison of patient characteristics between arms.

The survival parameters that will be considered are progression-free survival (PFS) and overall survival (OS). PFS is considered the time from the start of radiotherapy to the occurrence of progression or death. OS is considered the time from surgery to death (in relation to GBM). The Kaplan‒Meier method will be used to estimate the probability of survival. Survival comparisons between groups will be made using the log-rank or Gehan-Wilcoxon test. The Cox proportional hazards model will be used to calculate the hazard ratio in univariable and multivariable analyses.

All statistical tests and confidence intervals will be calculated at a significance level of 5%.

### Methods for any additional analyses {20b}

Not applicable. The subgroup and adjusted analyses are not planned.

### Definition of analyses population relating to protocol nonadherence and any statistical methods to handle missing data (multiple imputation) {20c}

Protocol nonadherence will be assessed by the principal investigator case by case. Patients with major protocol deviations will be excluded from the analysis. Missing data are not planned to be imputed. However, in the event of substantial missing data for any parameter, a sensitivity analysis using any method of imputation could also be used.

The data will be analysed for the individual groups below:

- Full Analysis Set (FAS)
  All patients who successfully completed recruitment met the inclusion/exclusion criteria.
- Per Protocol Set (PPS)
  FAS subgroup with such patients who undergo trial without significant deviations from the protocol.
- Safety Set
  Includes all patients who were administered a medicinal product within the framework of the GlioMET study.

## Methods: monitoring

### Data monitoring {21a}

The on-site monitoring of the study conduct is regularly performed throughout the study period to assess the accuracy and completeness of the data. The study site may be subject to a quality assurance visit. If so, the site will be contacted in advance to organize a monitoring visit. The investigator will ensure direct access to all study documents for quality assurance monitors.

### Interim analyses {21b}

Not applicable. An interim analysis is not planned.

### Harms {22}

Serious adverse events will be reported according to standard rules and standard operating procedures of the coordinating unit. All study participants will be monitored for potential adverse events.

### Auditing {23}

Trial conduct will be audited by the Institutional Projects Evaluation Committee every year during the study. The process of auditing will be independent of the investigator and sponsor.

### Research ethics approval {24}

This protocol and the template informed consent form were reviewed and approved by the Ethical Committee of the Masaryk Memorial Cancer Institute (in September 2020) with respect to the scientific content and compliance with applicable research and human subject regulations. Study protocol was evaluated by institutional Project Steering Committee and institutional Ethics Committee No. 2020/1206/MOU.

### Protocol Amendments {25}

Any new protocol modifications will be sent for review by the ethics committee and a national Drug Agency and will be amended at the clinical trial registry.

### Consent and Assent {26a}

Informed consent will be obtained from all participants prior to enrolment in the study.

A trained study investigator will describe the study to patients or authorized surrogates if applicable. Patients will also receive information sheets. The investigator will discuss the study with patients in the sense of the information provided in the information sheets. The investigator will obtain written informed consent from patients willing to participate in the trial. In the case of patients who are unable to consent because of a medical condition, their ability to participate in the study will be assessed by a medical council consisting of at least one independent physician informed about the study details and one study investigator.

### Confidentiality {27}

All study-related information will be stored securely at the study site. All participant information will be stored in locked file cabinets in areas with limited access. All local databases will be secured with password-protected access systems. Forms, lists, logbooks, appointment books, and any other listings that link participant ID numbers to add identifying information will be stored in a separate locked file in an area with limited access.

### Declaration of interest {28}

The authors declare that they have no competing interests.

### Access to data {29}

The final trial dataset will be accessible to all investigators participating in this study.

### Ancillary and posttrial care {30}

The clinical trial GlioMET is conducted in full accordance with the principles of the Declaration of Helsinki (in the latest valid version), in accordance with GCP requirements and in accordance with applicable national and European legislation. GlioMET was discussed and approved by the local ethics committee and State Institute for Drug Control before the start.

All patients were informed about the possible risks of their participation in this study and about the possibility of ending their participation in the study at any time without consequences. The patients are also informed about the measures that will be taken for the protection of personal data and about the fact that, in the event of damage to health as a result of the implementation of the study, liability insurance for the examiner and the contracting authority has been arranged in accordance with the applicable legislation, through which the possible compensation of assessment subjects.

### Dissemination policy {31a}

We intend to submit the results of the study to be published in a peer-reviewed international medical journal and disseminate them to academic and healthcare professionals via presentations at conferences.

## Data Availability

No datasets were generated or analysed during the current study.

## Abbreviations

2D-ROI: Two-dimensional region of interest
3D-ROI: Three-dimensional volume of interest
^11^C-methionine: 11 Carbon radiolabelled methionine
^18^F-FET: 18 Fluorine radiolabelled fluoroethyltyrosine
^18^F-FLT: 18 Fluorine radiolabelled fluorothymidine
^18^F-DOPA: 18 Fluorine radiolabelled dihydroxyphenylalanine
AA: Aminoacid
ALT: Alanine aminotransferase
AST: Aspartate aminotransferase
ATRX: Alpha-thalassemia X
CHT: Chemotherapy
D: Day
ECOG: Eastern Cooperative Oncology Group
eCRF: Electronic Case Report Form
EORTC: European Organization for Research and Treatment of Cancer
FAS: Full analysis set
FDG: 2-Deoxy-2-[18F] fluoro-D-glucose
FET: Fluoroethyltyrosine
Fri: Friday
GBM: Glioblastoma multiforme
GCP: Good clinical practice
GFAP: Glial fibrillary acidic protein
HBV: Hepatitis B virus
HCV: Hepatitis C virus
HIV: Human immunodeficiency virus
ID: Identificational data
IDH1: Isocitrate dehydrogenase 1
IDH2: Isocitrate dehydrogenase 2
IHC: Immunohistochemistry
IQR: Interquartile range
Ki-67 index: Marker of proliferation Kiel 67
M: Month
MET: Methionine
MEYS: Ministry of education, youth and spo
MGMT: O^6^-methylguanine-DNA methyl-transferase
miRNA: Micro-ribonucleic acid
MMCI: Masaryk Memorial Cancer Institute
Mon: Monday
MR: Magnetic resonance
MRI: Magnetic resonance imaging
OP: Overall survival
PD: Progressive disease
PDL1: Programmed Cell Death Ligand 1
PET/CT: Positron emission tomography/computer tomography
PFS: Progression-free survival
PoF: Patterns of failure
PPS: Per Protocol Set
preRT: Preoparative radiotherapy
PS: Performance status
QLQ: Quality of Life Questionnaire
RANO: Response Assessment in Neuro-Oncology
REP: Rapid early progression
RT: Radiotherapy
SUVavg: Average value of standardized uptake value
SUVmax: Maximum value of standardized uptake value
SD: Standard deviation
T/B ratio: Tumour to background ratio
TERT: Telomerase reverse transcriptase
TMZ: Temozolomide
ULN: Upper limit of normal
WHO: World Health Organization
